# Untargeted Lipidomics Study of Bipolar Disorder Patients in Serbia

**DOI:** 10.3390/ijms242216025

**Published:** 2023-11-07

**Authors:** Milka Jadranin, Nataša Avramović, Zoran Miladinović, Aleksandra Gavrilović, Ljubica Tasic, Vele Tešević, Boris Mandić

**Affiliations:** 1 University of Belgrade—Institute of Chemistry, Technology and Metallurgy, Department of Chemistry, Njegoševa 12, 11000 Belgrade, Serbia; milka.jadranin@ihtm.bg.ac.rs; 2 University of Belgrade—Faculty of Medicine, Institute of Medical Chemistry, Višegradska 26, 11000 Belgrade, Serbia; 3 Institute of General and Physical Chemistry, Studentski trg 12–16, 11158 Belgrade, Serbia; zmiladinovic@iofh.bg.ac.rs; 4 Special Hospital for Psychiatric Diseases “Kovin”, Cara Lazara 253, 26220 Kovin, Serbia; gavrilovicaleksandra74@gmail.com; 5 Institute of Chemistry, Organic Chemistry Department, State University of Campinas, Campinas 13083-970, Sao Paulo, Brazil; ljubica@unicamp.br; 6 University of Belgrade—Faculty of Chemistry, Studentski trg 12–16, 11000 Belgrade, Serbia; vtesevic@chem.bg.ac.rs

**Keywords:** bipolar disorder, biomarkers, lipidomics, serum, mass spectrometry

## Abstract

The Lipidomic profiles of serum samples from patients with bipolar disorder (BD) and healthy controls (C) were explored and compared. The sample cohort included 31 BD patients and 31 control individuals. An untargeted lipidomics study applying liquid chromatography (LC) coupled with high-resolution mass spectrometry (HRMS) was conducted to achieve the lipid profiles. Multivariate statistical analyses (principal component analysis and partial least squares discriminant analysis) were performed, and fifty-six differential lipids were confirmed in BD and controls. Our results pointed to alterations in lipid metabolism, including pathways of glycerophospholipids, sphingolipids, glycerolipids, and sterol lipids, in BD patient sera. This study emphasized the role of lipid pathways in BD, and comprehensive research using the LC-HRMS platform is necessary for future application in the diagnosis and improvement of BD treatments.

## 1. Introduction

Bipolar disorder (BD) is a psychiatric illness defined by altering mood states such as euthymia, major depression, and mania [1]. BD is correlated with impaired quality of life, disability, and premature mortality, with a prevalence of 60 million people worldwide [2]. Diagnosis of BD exclusively depends on the subjective recognition of symptoms, still without objective methods such as a clinical test of biomarker identification, instigating misdiagnosis, inadequate treatments, and deficient clinical outcomes [3,4,5,6]. Mainly, BD patients have a depressive episode in the initial phase of the disease, whose symptoms slightly differ from unipolar depression [7], and often, only some BD cases are correctly diagnosed and treated, causing the progression of illness [1,8,9]. Additionally, current antipsychotics and antidepressants used in treatments are effective in only 40–60% of BD patients, causing severe side effects [10,11]. Therefore, discovering potential biomarkers is crucial to providing accurate and early diagnosis and monitoring of BD [12]. Desirable biomarker candidates should have features including their correlation with functional outcomes and practical, inexpensive, reproductive, and non-invasive methods of their determination and monitoring [13,14]. Currently, proteomics, genomics, and metabolomics are important platforms for measuring potential biomarkers in psychiatric diseases [15,16,17,18,19,20,21,22,23]. In the near future, combined data obtained by all ‘omics’ platforms in predictive models might provide a comprehensive view of BD diagnosis [14,24,25].

Lipids include different subclasses that play an important role in the regulation of neuronal development and function, modulation of neuronal plasticity of membranes [26,27,28], as well as the effect on energy metabolism in the brain [29,30,31]. Lipidomics is not a sufficiently explored ‘omics’ platform in the study of psychiatric diseases [32,33,34], although the recent comprehensive identification of hundreds of different lipid molecules in tissues, plasma, and serum using liquid chromatography coupled to mass spectrometry confirms its important role in the biomarker discovery of psychiatric diseases [35,36,37]. Alterations in the lipid metabolism were described, including changes in free fatty acid (FFA), glycerophospholipids (GP), sphingolipids (SP), and glycerolipids (GL) in plasma and serum [38,39,40,41,42,43,44,45], as well as in the arachidonic acid metabolism in the brain and periphery [37,46,47].

The objective of the present study was to carry out an untargeted lipidomics of serum samples from BD patients and healthy individuals to examine their lipid profiles and to analyze differential lipids in these two groups. These results might provide comprehensive insights into alterations in lipid metabolism in BD and the identification of potential biomarkers, improving further studies on the diagnosis and treatment of BD.

## 2. Results

A total of 31 patients with BD, including 13 males and 18 females, with ages between 20 and 74 years, and 31 healthy control participants, males (16) and females (15), with ages between 24 and 54 years old, were included in this study (Table 1). There are no statistically significant differences in age, gender, or BMI (body mass index) between the groups. Blood serum samples from both groups were taken after a minimum of 8 h of fasting and prepared in triplicate for further analysis.

### Selection of Potential Lipid Biomarkers

**Figure 1:**
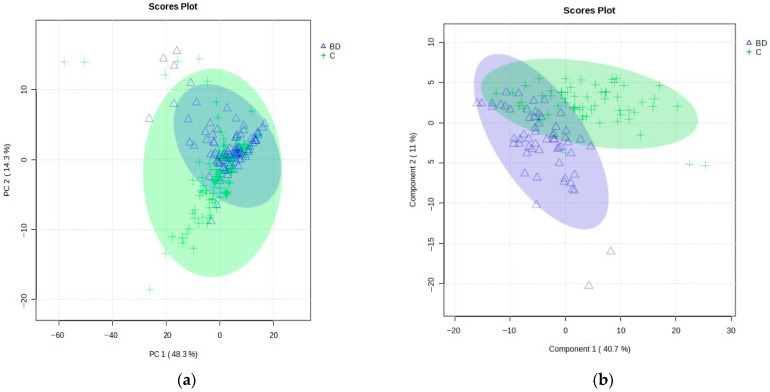
(**a**) PCA model scores plot: PCs 1 and 2 (with 48.3% and 14.3% variance, respectively) of the MS data obtained for all BD patients (blue, triangles) and healthy controls (C, green, crosses), showing 95% confidence ellipses for these two groups. (**b**) Results of the PLS-DA model score plot of the MS data obtained for the training set of BD patients (blue, triangles) and healthy controls (C, green, crosses) using five components, with 40.7% variance in Component 1 and 11.0% in Component 2.

A total of 201 *m*/*z* features for combined negative–positive ion modes were selected using the LC–HRMS method in this study. Principal component analysis (PCA) was performed on all samples, including BD patients and healthy controls (C), to observe the natural tendency of grouping into the classes of interest and to identify anomalous samples (outliers). As can be seen from Figure 1a, the PCA results confirmed no clear separation between BD and C and greater homogeneity among the BD samples. The total variance explained by the five components (all constructed with 201 variables) of the PCA model was 74.9%, whereas the first two PCA components accounted for 48.3% and 14.3%, respectively. The number of components and PCA model were determined using the Scree plot and the number of captured variances for each component from MetaboAnalyst.

Overall, 12 outliers (5 BD samples of 3 patients and 7 C samples of 4 individuals) were identified by PCA as samples lying outside the corresponding 95% Hoteling’s T2 confidence ellipse.

After removing identified outliers and before further proceeding to assemble supervised-based models, the initial data set was split into training and test parts, as explained in the experimental section. Then, the supervised models on the training set of samples (118 samples: 59 BD and 59 C) were applied to explore the significant features that differentiated the groups, considering the variable importance in projection (VIP) scores with values higher than 1.0. The results of the partial least squares discriminant analysis (PLS-DA) are shown in Figure 1b.

**Figure 2:**
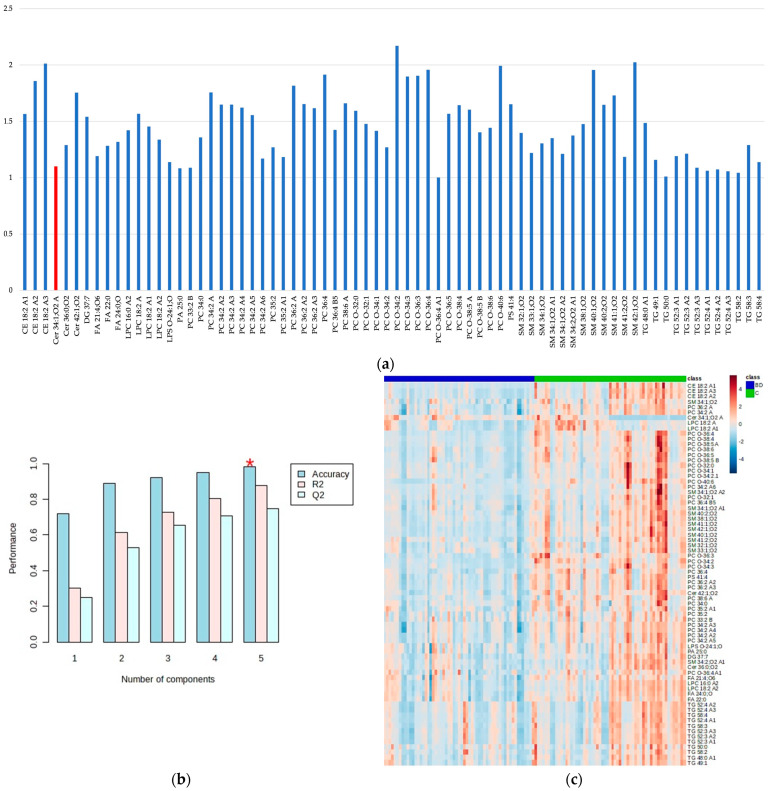
The variable importance in projection (VIP) values greater than 1.0 obtained by the PLS-DA model show the most important metabolites that are discriminatory for BD patients and healthy controls (C); the metabolites more abundant in C samples are shown in blue, while the metabolite more abundant in BD samples is shown in red (**a**), the PLS-DA classification using five components (the red star indicates the best classifier). Accuracy: 0.983, R^2^: 0.878, and Q^2^: 0.748 (**b**), and cluster analysis obtained with the top 72 variables of VIP scores generated by the PLS-DA presented as a heatmap (distance measure using Euclidean) and clustering algorithm using ward (D). The BD samples are shown in blue and C in green (**c**).

The PLS-DA score plot showed the observed clustering tendency between the BD and C groups. The *m*/*z* values of lipids that distinguished BD from C samples were revealed by validated PLS-DA classification using five components (accuracy: 0.983, R^2^: 0.878, and Q^2^: 0.748), and among them, 72 differential *m*/*z* features belonging to the 56 lipid molecules (Figure 2a and Table 2) showed the highest contributions to group separation, with VIP values higher than 1.0. The lipids were tentatively assigned using the accurate mass measurements and databases—LIPID MAPS Structure Database (LMSD) (https://www.lipidmaps.org/, accessed on 21 August 2023) [48] and Human Metabolome Database (HMDB) (https://hmdb.ca/, accessed on 21 August 2023) [49] (Figure 2a and Table 2). The cluster analysis presented in the heatmap (Figure 2c) also confirmed the clustering tendency of groups.

**Figure 3:**
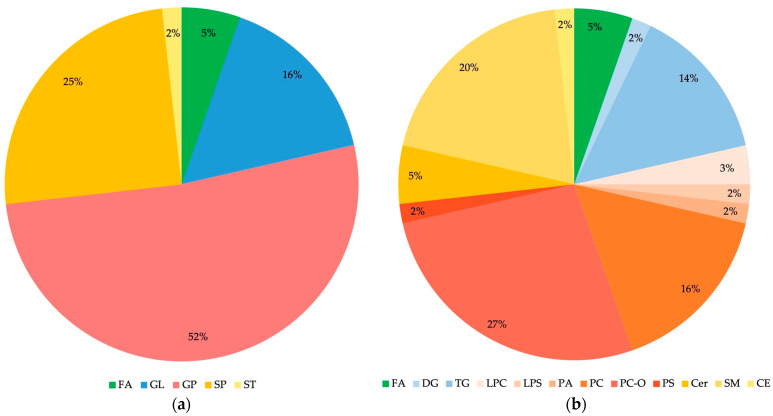
Distribution of lipid classes (**a**) and subclasses (**b**) in differential lipids identified for BD and C comparison. FA: fatty acyls; GL: glycerolipids; GP: glycerophospholipids; SP: sphingolipids; ST: sterol lipids; DG: diacylglycerols; TG: triacylglycerols; LPC: 1-acyl-sn-glycero-3-phospho-cholines; LPS: 1-acyl-sn-glycero-3-phosphoserines; PA: 1,2-diacyl-sn-glycero-3-phosphates; PC: diacylglycerophosphocholines; PC O-: 1-alkyl,2-acyl-glycerophosphocholines; PS: diacylglycerol-phosphoserines; Cer: N-acylsphinganines (dihydroceramides); SM: ceramide phosphocholines (sphingomyelins); CE: cholesterol ester. Identified Cer, FA, LPC, PC, PC O- and TG contain saturated as well as unsaturated fatty acids; PA contains only saturated fatty acids, while CE, DG, LPS, PS, and SM contain only unsaturated fatty acids.

Almost all lipid molecules show the highest contributions to group separation, with VIP values higher than 1.0, with the exception of Cer 34:1;O2 and are more abundant in C samples. These lipids are typically rich in double bonds, i.e., unsaturated fatty acids (UFAs, one double bond) and polyunsaturated fatty acids (PUFAs, multiple double bonds, up to six in PC 38:6, PC O-38:6, and PC O-40:6). The lipid molecules that contribute the most to the separation of BD and C groups (Figure 2a and Table 2) belong to different lipid classes, including: (1) GP (mainly 1-alkyl,2-acyl-glycerophosphocholines (PC O-) and diacylglycerophosphocholines (PC), and a small number of 1-acyl-sn-glycero-3-phosphocholines (LPC), 1-acyl-sn-glycero-3-phosphoserines (LPS), 1,2-diacyl-sn-glycero-3-phosphates (PA), and diacylglycerol-phosphoserines (PS)), (2) SP (mainly ceramide phosphocholines (sphingomyelins) (SM) and some *N*-acylsphinganines (dihydroceramides) (Cer)), and (3) GL (mainly triacylglycerols (TG)). In contrast, fatty acyls (FA) and sterol lipids (ST)—cholesterol ester (CE)—are much less represented. Details about the distribution of lipid classes in percentages are given in Figure 3.

**Figure 4:**
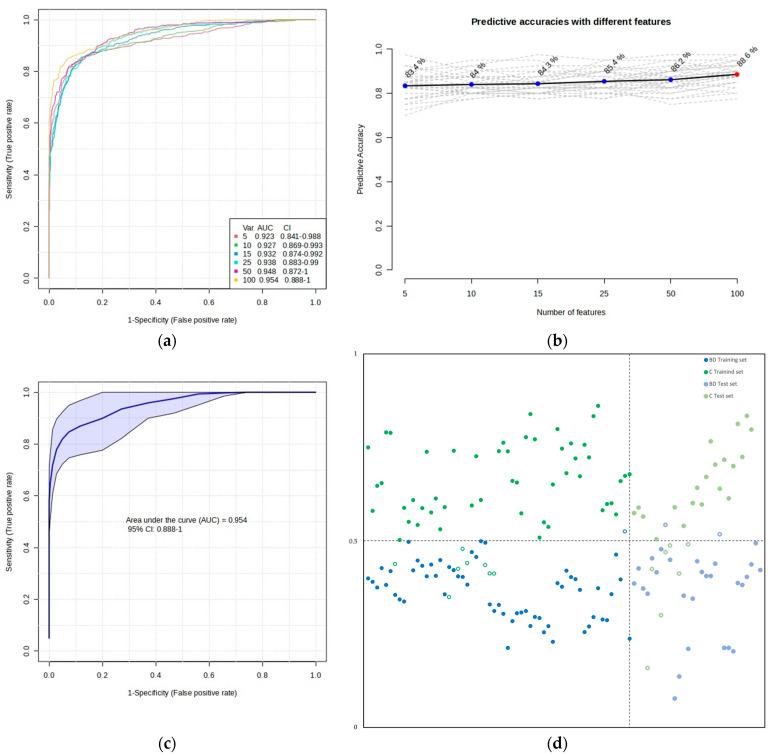
Biomarker prediction and validation by multivariate ROC curve-based exploratory analysis and multivariate ROC curve-based model evaluation (Tester), respectively. (**a**) Overview of all ROC curves created by MetaboAnalyst 5.0 from six different biomarker models considering different numbers of features (5, 10, 15, 25, 50, and 100) with their corresponding AUC and CI values. The ROC test classification method was PLS-DA, and the feature ranking method was PLS-DA built-in with two latent variables. (**b**) Graphic presenting the predictive accuracies of six different biomarker models with an increasing number of features. The red dot specifies the highest accuracy for the 100-feature panel of Model 6. (**c**) ROC curve for selected biomarker Model 6. (**d**) The predicted class probabilities of the training set of samples using selected biomarker Model 6 (accuracy: 92.4%; sensitivity: 98.3%; specificity: 86.4%)—on the left side, and the predicted scores for the test set of samples (accuracy: 83.9%; sensitivity: 79.4%; specificity: 90.9%)—on the right side. The classification boundary is located at the center (x = 0.5, the dotted line). Misclassified samples in both training and test sets are shown as empty circles, blue for the BD group and green for the C group.

To obtain potential biomarkers, multivariate ROC curve exploration analysis has been performed using PLS-DA as the classification method and PLS-DA built-in with two latent variables as the feature ranking method (Figure 4). All ROC curves created by MetaboAnalyst 5.0 from six different biomarker models considering different numbers of features (5, 10, 15, 25, 50, and 100) presented the area under the curve (AUC) at least 0.923 with a confidence interval (CI) between 0.841 and 1.000 (Figure 4a). The highest accuracy was achieved for the 100-feature panel of Model 6 (Figure 4b), which presented AUC = 0.954 (0.888–1.000 with 95% CI) (Figure 4c), accuracy 92.4%, sensitivity 98.3%, and specificity 86.4% (Figure 4d, left side).

To estimate the validity of the model created with the training set of samples, ROC curve-based model evaluation (Tester) analysis (using the PLS-DA algorithm with two latent variables) was performed with the test set of samples (consisting of a total of 56 samples: 29 BD and 27 C). The results of this analysis are presented in Figure 4d on the right side. There, the average of the predicted class probabilities of samples across the 100 cross-validations using the created classifier is shown. As the algorithm uses a balanced sub-sampling approach, the classification boundary is located at the center (x = 0.5, the dotted line).

## 3. Discussion

Based on our knowledge, only several plasma and serum lipidomics studies of BD applying LC coupled to MS are known so far [39,40,41,42,43,44,45,50]. In this study, we explored an untargeted analysis of extracted lipids from the serum samples of Serbian BD patients and healthy controls (C) to reveal statistically important differences in their lipid profiles with the main aim of identifying potential biomarker candidates for BD. Our results indicated five of the most affected lipid classes: GP, SP, GL, FA, and ST, with different distributions in the serum lipid profiles of the BD and C groups (Figure 2a and Table 2). GPs are the most abundant among differential lipids, with the dominant prevalence of PC-O and PC subclasses and significantly less presence of PA, PS, LPS, and LPC subclasses, as displayed in Table 2 and Figure 2 and Figure 3. SPs include the dominant presence of SM and significantly less presence of Cer subclasses, while GLs consist of dominant TG and negligible presence of DG lipids (Figure 2 and Figure 3). All these lipid classes have decreased concentrations in BD patients compared to controls, except in SPs’ subclass of ceramides, which corresponds to Cer 34:1;O2, with increased concentrations in BD patients compared to controls (Figure 2 and Figure 3). Accordingly, Schwarz et al. (2008) reported that white matter from postmortem brain tissue revealed increased levels of C36:1, C36:2, and C34:1 ceramides compared to controls [38]. Brunkhorst-Kanaan et al. (2021) also documented increased concentrations of C16:0, C18:0, C20:0, C22:0, C24:0, C24:1 ceramides, glucosylceramide C24:1, and lactosylceramide C24:0 in the serum of BD patients compared to controls [50]. This research group suggested aging as a risk factor for BD because the increased level of long-chain ceramides is positively correlated with age. Moreover, Li et al. (2023) recently conducted a multi-omics analysis of the medicament-induced model of bipolar disorder in zebrafish to explore the molecular mechanism of altered metabolic pathways in this disease [51]. Their lipidomics study showed increased levels of FA, SP (SM, glycosylceramides (GlyCer)), and GP (PC, PE, LPC), while Cer was significantly reduced in the brains of the BD group compared to the controls. These results were explained by the lack of conversion of FA to Cer and further transformation to SM and GlyCer in SP metabolism. The decreased level of ceramides agrees with our results regarding the majority of ceramides except for one subclass, Cer 34:1;O2.

Ribeiro et al. (2017) demonstrated that treated patients with BD type I exhibited decreased levels of GP in serum compared to the healthy control (HC) group, although they increased levels of SP and GL in BD, which is contrary to our results [44]. Knowles et al. (2017) identified a peripheral biomarker, serum-based phosphatidylinositol, which shows a significant correlation with BD risk [52]. This research group confirmed a decrease in GP levels compared to controls, which is also in agreement with our results [52]. Guo et al. (2022) recently reported the quantitative analysis of plasma lipid composition in adult women with BD and controls (HCs), indicating decreased levels of phosphatidylethanolamines, phosphatidylserine, and SM in BD compared to HC, which reflects a negative correlation of these lipids with the severity of psychotic, affective, or mania symptoms [39,40,53,54]. Previously reported research also showed that plasma SM has an inverse correlation with depressive symptoms [55]. Costa et al. (2023) recently explored the lipidomic profiles of plasma samples from drug-naïve patients in the schizophrenia group (SZ), bipolar disorder (BD), and healthy control groups and compared them crosswise [45]. Their results confirmed alterations in lipid pathways, such as the metabolism of GP, SP, and GL. When the lipidomic profiles of plasma BD and control groups were quantitatively compared, increased levels of GL and SP, mostly SMs, as well as some species of GP and ST, were identified. These results are not in agreement with our results, as well as with the majority of reported studies, and they were explained by the early stages of the disease in the patients’ samples [45].

Lipids have important multifunctional biological functions, including structural integrity and fluidity of membranes and transmembrane signaling in the brain that are closely correlated to the processes of inflammation, apoptosis, proliferation, and differentiation [56,57], and dysfunction of lipid metabolism has been emphasized in psychiatric disorders, including BD [39,58]. The majority of lipid molecules implicated in SP (SM, Cer) and GP (PE, PC-O, PC, LPC, and LPE) metabolism were reduced, but only one ceramide subclass (Cer 34:1;O2) involving the SP metabolism was enhanced in the serum of the BD group relative to the control group. SPs and GPs are the main components of brain membranes. While SMs play a crucial role in transmembrane signaling, GPs (PE, PC, PC-O, LPC, and LPE) have a major role in neuronal membrane integrity and fluidity [52,59]. Our results obviously pointed to the alteration in GP and SP pathways, indicating disruption of lipid homeostasis in the brain, including alteration of membrane structure and intracellular signaling pathways that are responsible for the pathology of BD.

**Figure 5:**
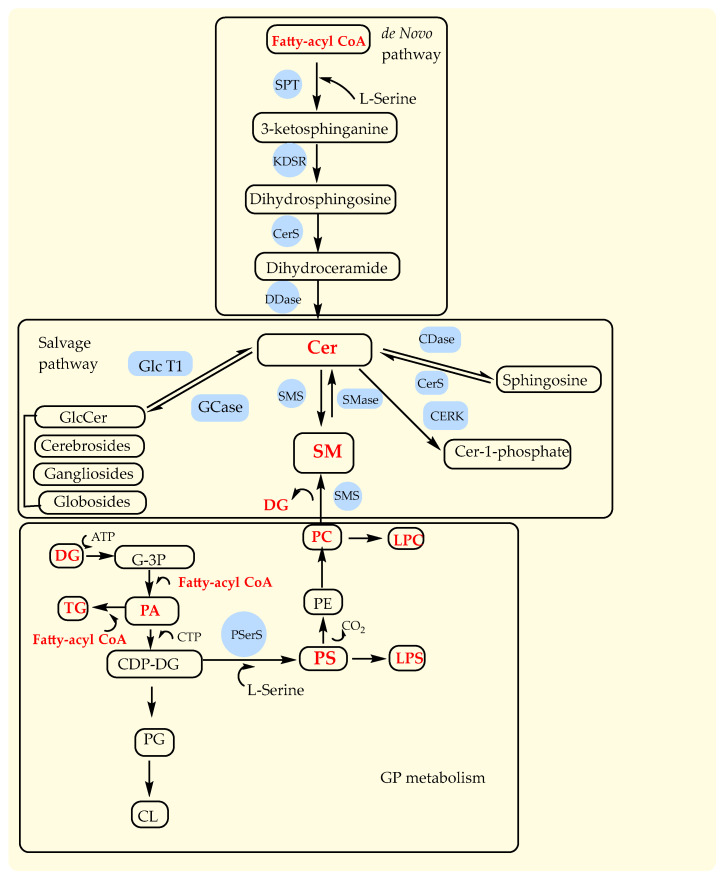
Major pathways in sphingolipid metabolism (de novo and salvage pathways) and glycerophospholipid metabolism. Cer, ceramides; SM, sphingomyelins; PC, phopshatidylcholine; PE, phopshatidylethanolamine; PS, phosphatidylserine; LPS, lysophosphatidylserine; LPC, lyso-phosphatidylcholine; DG, diacylglicerols; TG, triacylglicerols; G-3P, glycerol-3-phosphate; PA, phosphatidic acid; CDP-DG, cytidine diphosphate-diacylglycerol; PG, phosphatidylglycerol; CL, cardiolipin. Enzymes: SPT, serine palmitoyltransferase; KDSR, NADH-dependent 3-keto-sphinganine reductase; CerS, ceramide synthases; DDase, dihydroceramide desaturase; CDase, ceramidase; SMase, sphingomyelinase; SMS, sphingomyelin synthase; GlcT-1, glucosylceramide synthase.

SP metabolism provides the synthesis of many important lipid molecules catalyzed by numerous enzymes through either de novo or the salvage pathways [59]. De novo SP metabolism starts first with the condensation of L-serine and a fatty-acyl CoA (palmitoyl-CoA, myristoyl-CoA, or stearoyl-CoA) producing 3-ketosphinganine, which is then reduced to dihydrosphingosine and further acylated to dihydroceramide (Figure 5). Finally, dihydroceramide is desaturated to ceramide and is also produced by salvage pathway by decomposition of complex glycolipids, hydrolysis of SMs, and acylation of sphingosine (Figure 5). Ceramides are also correlated with GP metabolism because, in the reaction with PC catalyzed by the enzyme sphingomyelinase (SMase), they are transformed into SMs and DG. Actually, GP metabolism begins with glycerol-3-phosphate and fatty-acyl CoA, as well as DG and fatty-acyl CoA, to produce PA, which is further transformed into all other GP species, such as PS, PE, PC, LPC, and LPS (Figure 5). Our results identified that Cer, FA, LPC, PC, PC O-, and TG contain saturated and unsaturated fatty acids; PA contains only saturated fatty acids; and DG, LPS, PS, and SM contain only unsaturated fatty acids. Therefore, there is probably a defect in the transformation of DG with fatty-acyl CoA to the PA and further on to other phospholipids (PC, PS, LPS, and LPC) in GP metabolism. Also, the increased level of only Cer 34:1;O2 species from all GP classes indicated increased activity of ceramide synthases (CerS) that catalyze the acylation of dihydrosphingosine to dihydroceramide, especially its CerS5 and six isoforms that have specificity for the fatty-acyl C14–C16 CoA [59] (Figure 5). Ceramides are like the second messenger, with the important role of regulating the activity of numerous proteins, among which is phospholipase A2 [59].

Our results obviously pointed to the alteration of glycerophospholipid metabolism – GP, sphingolipids – SP, and glycerolipids – GL pathways, which might be correlated with the alteration of enzyme phospholipase A2 (PLA2) activity, which is responsible for the decomposition of glycerophospholipids into fatty acids. It has been shown that PLA2 activity is increased in schizophrenia (SCZ) and BD with ultra-high risk for psychosis patients, and it is correlated with changes in neuronal function contributing to affective and cognitive symptoms [45]. Putative lipid biomarkers determined in our lipidomics study were considered through GP and SP metabolic pathways with the aim to indicate their importance as well as the enzymes they regulate to emphasize the possibility of their consideration in further studies of a universal set of biomarkers.

At last, but not at least, there are some limitations of this study, as it is an untargeted lipidomics study applying liquid chromatography coupled with high-resolution mass spectrometry (LC-HRMS) that was carried out to accomplish lipidomic profiles of serum samples from patients with BD and healthy controls. Firstly, the validation of potential biomarkers was not done with an independent group. Secondly, the obtained results were not acquired by a fully quantitative and validated method. Thirdly, the structures of putative biomarkers were not confirmed using reference material. Fourthly, the BD patients included in this study were under medical therapy of antipsychotics of the first and the second generation.

## 4. Materials and Methods

### 4.1. Sample and Sample Preparation

This study was approved by the Ethics Committee of the Special Hospital for Psychiatric Diseases “Kovin,” the University of Belgrade—Faculty of Chemistry, and the Blood Transfusion Institute of Serbia. Blood samples of selected BD patients under medical treatment were obtained from the Special Hospital for Psychiatric Diseases “Kovin,” while samples of healthy controls were provided by the Blood Transfusion Institute. All participants or their caretakers provided written consent before their enrollment in this study. A total of 31 BD patients, including 13 males and 18 females, with ages between 20 and 74 years, were analyzed in this study. The control group comprised 31 healthy volunteers, males (16) and females (15), with ages between 24 and 54 years old. A total of 2 patients were using antipsychotics of the first generation (chloropromazine, levomepromazine), and 24 patients were using antipsychotics of the second generation (aripiprazole, clozapine, quetiapine, olanzapine, risperidone), 5 patients were using anxiolytics (alprazolam, clonazepam, diazepam, lorazepam, prazepam), and healthy controls were under no medical therapy. Blood samples were collected from the patients and healthy controls in the morning hours, before the first meal. The blood samples were kept on ice for one hour, centrifuged, and then the sera collected from the supernatants were stored at −80 °C. The maximum period of storage before analysis was up to two weeks.

### 4.2. Chemicals

Chloroform (for HPLC, >99.8%, amylene stabilized, Sigma-Aldrich, France), methanol (LC–MS, Chromasolv™, ≥99.9%, Honeywell, Germany), 2-propanol (LiChrosolv^®^, hypergrade for LC–MS, Merck, Darmstadt, Germany), acetonitrile (LiChrosolv^®^, hypergrade for LC–MS, Merck, Darmstadt, Germany), and deionized water (18.2 MΩcm^−1^, Barnstead™ Smart2Pure™ Water Purification System, Thermo Scientific™, USA) were used for lipids’ extraction, dissolution, and preparation of the mobile phases for the LC–HRMS analyses. Ammonium formate (puriss. *p.a.*, eluent additive for LC–MS, Fluka, USA), formic acid (eluent additive for LC–MS, Fluka Analytical), and ammonium acetate (Optima^®^ LC/MS, Fisher Chemical, USA) were used for the preparation of eluent additives for LC–HRMS.

### 4.3. Lipidomics Analysis

#### 4.3.1. Lipid Extraction from Blood Serum Samples

Lipids were extracted from serum samples according to the methodology described by O’Brien et al. [60]. A total of 100 μL of serum sample thawed on ice were transferred to a 2.0 mL Eppendorf tube, and 100 μL of chloroform and 100 μL of methanol, both ice cold, were added. This mixture was mixed (vortex, MX-S) for 2 min, left to stand at −4 °C for 30 min, and then centrifuged for 15 min at 15,000 g (DLAB Centrifuge D2012 Plus) to yield the upper (methanol/aqueous fraction) and lower phases (chloroform/lipid fraction). After centrifugation, 75 μL aliquots of the lower phase, including lipids and chloroform, were carefully sampled into 1.5 mL Eppendorf tubes, and the solvent was evaporated till dryness in the mild nitrogen stream. The remaining components were resuspended in 1 mL of the mixture 2-propanol—acetonitrile—deionized water (2:1:1, *v*/*v*/*v*) at room temperature, transferred into 2 mL glass vials, and subjected to the LC–HRMS analysis. To detect possible sources of instrumental variation in the batch analysis of the samples, a third group of samples was analyzed, named quality control (QC), consisting of a pool of all samples (BD and HC), prepared following the same procedure using a pooled serum sample. Solvent blank samples, prepared following the same procedure using deionized water, were injected at the beginning and at the end of each batch to monitor background signals and contamination. A personal computer system running Agilent MassHunter software (revisions B.06.01 and B.07.00) was used for data acquisition and processing, respectively. All serum samples were prepared in triplicate.

#### 4.3.2. Liquid Chromatography–High-Resolution Mass Spectrometry (LC–HRMS) Measurements

Untargeted lipidomics was performed by injection of prepared samples into the same analyzing system used in our previous studies [61,62], consisting of a liquid chromatograph (1290 Infinity LC system; Agilent Technologies, Waldbronn, Germany) with a quaternary pump, a column oven, and an autosampler connected to the Quadrupole Time-of-Flight mass detector (6550 iFunnel Q-TOF MS, Agilent Technologies; Santa Clara, CA, USA) equipped with a dual spray Agilent Jet Stream (AJS) electrospray ion source. Separation of lipid compounds was carried out using a Zorbax Eclipse Plus C18 column RRHD (100 mm × 2.1 mm; 1.8 μm, Agilent Technologies). The mobile phase was composed of solvents A: water/ACN (40:60, *v*/*v*) and B: IPA/ACN (90:10, *v*/*v*); both solvents contained 10 mmolmL^−1^ ammonium formate and 0.1% formic acid (positive ionization mode) or 10 m mmolmL^−1^ ammonium acetate (negative ionization mode). The following gradient program was used: 0–2 min 15–30% B, 2–2.5 min 30–48% B, 2.5–8.5 min 48–72% B, 8.5–11.5 min 72–99% B, 11.5–12 min 99% B, 12.0–12.1 min 99–15% B, 12.1–15 min 15% B. The mobile phase flow rate was 0.60 mLmin^−1^, the column temperature was 60 °C, and the injection volume of samples and blanks was 2 μL (positive ionization mode) or 4 μL (negative ionization mode). After separation, the lipids were analyzed using a mass detector. Positive and negative ion modes were recorded separately. The instrument was operated in MS mode in the *m*/*z* range of 80–1700 under the following conditions: capillary voltage, 3500 V, fragmentor voltage, 175 V, nozzle voltage, 1000 V, skimmer 1, 65 V, octupole RF peak, 750 V, desolvation gas (nitrogen) temperature, 200 °C, desolvation gas (nitrogen) flow, 14 Lmin^−1^, nitrogen flow, 11 Lmin^−1^. Ions *m*/*z* 121.0508 and 922.0097 in positive ion mode and 112.9855, 966.0007, and 1033.9881 in negative ion mode were used as a lock mass for accurate mass measurements. Samples were recorded in consecutive batches for both positive and negative ionization modes, always after cleaning the ion source. To reduce the impact of small variations in instrument sensitivity during the measurements, samples were randomly analyzed, and QC samples, prepared by pooling aliquots from all serum specimens, were injected before the first and after every nine injections of lipid extract samples to monitor the system stability. Solvents blank samples were injected at the beginning and end of each batch to monitor background signals and contamination. A personal computer system running Agilent MassHunter software (revisions B.06.01 and B.07.00) was used for data acquisition and processing, respectively.

### 4.4. LC–HRMS Data Processing and Statistical Analysis

The raw data (d) were converted to *mz*Data format for peak picking using Agilent MassHunter software (revision B.07.00). Peak detection and retention-time alignment were performed using the XCMS online platform within the R statistical programming environment [63,64,65].

For the collected data, optimized XCMS parameters include centwave feature detection, orbiwarp retention time correction, a minimum fraction of samples in one group to be a valid group = 0.50, *p*-value thresholds for patients versus control samples = 0.05, isotopic ppm error = 15, the width of overlapping *m*/*z* slices (mzwid) = 0.015, bandwidth grouping (bw) = 5, minimum peak width = 5 s, maximum peak width = 20 s. Thus, the raw data table of retention times, *m*/*z* values, and peak intensities were exported for further processing—cleaning of background noise, isotopic, and unrelated ions according to the data obtained by the Molecular Feature Extraction (MFE) tool in the MassHunter Qualitative Analysis Software (revision B.07.00, Agilent Technologies). For data extraction, the pre-set “small molecules (chromatographic)” algorithm was applied, with 200 counts as the limit for the background noise. In addition, the adduct settings (H^+^, Na^+^, K^+^, neutral loss of water, and NH_4_^+^) for positive ionization were applied due to the ammonium formate in the mobile phase, and (H^−^, CH_3_COO^−^) for negative ionization due to the ammonium acetate in the mobile phase. The option for “salt-dominated ion” was also applied.

The impact of possible small variations in instrument sensitivity during the measurements was checked by intraday and interday CV for the filtered *m*/*z* (rt) values in the QC samples. Since their coefficients of variation (CV) were under 30%, variations in instrument sensitivity were considered not to affect the results, so there was no need for further normalization of the samples.

The resulting table of retention times, *m*/*z* values, and peak intensities was organized into a single matrix containing the samples (cases) in the rows and the *m*/*z* (rt) values in the columns (variables), with additional categorical variables for rows referring to the classification of the samples (class variable: BD—bipolar disorder patients and C—healthy controls). The initial data set matrix was constructed with 186 chromatograms (93 for the BD group and a total of 31 patients, and 93 for the C group—31 individuals) and 201 variables for combined negative–positive ions and analyzed by principal component analysis (PCA) using the MetaboAnalyst 5.0 software platform (Xia Lab, McGill University, Montréal, QC, Canada, https://www.metaboanalyst.ca/, accessed on 10 August 2023) [66]. This unsupervised analysis was performed using the five principal components for discrimination of all analyzed samples, using no further data filtering, no data normalization, no data transformation, and autoscaling (mean-centered and divided by the standard deviation for each variable) for data pre-processing.

After the removal of outlier samples identified by PCA (5 BD samples from 3 individuals and 7 C samples from 4 individuals), as those were found outside the corresponding 95% Hoteling’s T2 confidence ellipse, the initial data set was randomly divided into two sets: the training set, which accounted for approximately 2/3 of the complete number of samples in the data table, keeping the same ratio of the number of class samples as an initial data set (stratify option). The test data set was used for external validation of the PLS-DA and the ROC models that incorporated the remaining (approximately 1/3 of the complete) samples of the initial data set. As a result of the splitting procedure, the training data set consisted of 118 total samples: 59 BD samples from 21 individuals and 59 C samples from 20 individuals, whereas the test data set included 56 samples, among which 29 were BD samples from 10 individuals and 27 C samples from 10 individuals. All samples in both data sets are grouped as triplicates for each patient, and their allocation inside the data set was assigned to a specific categorical variable [19].

The supervised Partial Least Squares Discriminant Analysis (PLS-DA) was performed on the training data set using the MetaboAnalyst 5.0 software platform (Xia Lab, McGill University, Montréal, QC, Canada, https://www.metaboanalyst.ca/) [66]. For discrimination of these samples, the five principal components were used, with no further data filtering, no data normalization, no data transformation, and autoscaling (mean-centered and divided by the standard deviation for each variable) for data pre-processing. A 10-fold CV was applied as the cross-validation (CV) method, and accuracy and variable importance in projection (VIP) were used to measure performance and importance features, respectively.

The lipid molecules were assigned based on accurate mass measurements and databases—LIPID MAPS Structure Database (LMSD) (https://www.lipidmaps.org/, accessed on 21 August 2023) [48] and Human Metabolome Database (HMDB) (https://hmdb.ca/, accessed on 21 August 2023) [49].

### 4.5. Identification of Potential Metabolite Biomarkers

For biomarker prediction, evaluation, and validation, the multivariate receiver operating characteristic (ROC) tests on the training and test data sets, respectively (with all 201 variables), were performed in MetaboAnalyst 5.0 (Xia Lab, McGill University, Montréal, QC, Canada, https://www.metaboanalyst.ca/) [66]. Specifically, for biomarker prediction, applied analysis—multivariate exploratory ROC curve analysis (Explorer) performs automated important feature identification and performance evaluation. The ROC test classification method was PLS-DA, the feature ranking method was PLS-DA built-in with two latent variables, and the ROC plots were generated by Monte Carlo cross-validation (MCCV) using balanced sub-sampling. In each MCCV, two-thirds (2/3) of the samples are employed to evaluate the feature’s importance. The top important features (2, 3, 5, 10, …, 100 as a maximum) are then exploited to build the classification models, which are validated on the remaining 1/3 of the samples. The procedure is replicated multiple times to calculate the performance and the confidence interval of each model. Multiple algorithms are available for classification and feature ranking methods. For our data, the classification method selected was PLS-DA, and the feature ranking method selected was the PLS-DA built-in algorithm with two latent variables (LV) [67].

For the evaluation and validation of the created ROC curve-based model, the PLS-DA algorithm with two latent variables was used. Samples from the test subset were excluded for external validation purposes. To obtain a decent ROC curve for the validation, the test set contained a balanced number of samples from both groups, as recommended (Xia Lab, McGill University, Montréal, QC, Canada, https://www.metaboanalyst.ca/) [66].

## 5. Conclusions

Although the application of MS-based untargeted lipidomics in the BD study is still mostly unknown, alterations of glycerophospholipids, sphingophospholipids, and glycerolipids indicated an important role of lipid pathways in the pathogenesis of bipolar disorder. The BD-treated patient’s lipid metabolism was definitively altered, with decreased levels of the majority of the most affected lipid classes: glycerophospholipids (GP), sphingolipids (SP), glycerolipids (GL), sterol lipids (ST), and fatty acyls (FA). GPs are the most abundant among differential lipids. From GPs, only one ceramide subclass, Cer 34:1;O2, which is involved in the SP metabolism, was increased in the serum of the BD group relative to controls. The increased level of Cer 34:1;O2 species demonstrated enhanced activity of ceramide synthases (CerS), actually CerS5 and six isoforms that have specificity for the fatty-acyl C14–C16 CoA. Ceramides also indicated probably increased activity of phospholipase A2, responsible for the decomposition of glycerophospholipids into fatty acids and their transformation into other lipids. Our results of the serum lipidomic profile of BD patients were mostly in agreement with previously published lipidomics BD data. To achieve a universal set of biomarkers, the key requirement is to explore a consolidated analysis of serum and plasma samples of different geographical and ethnic origins, including larger sample sizes and a wider age range, as well as samples of drug-naïve BD patients, to remove doubts about which biomarkers are the result of the disease and which are the result of drugs used in medical treatment. A detailed investigation of the biochemical alterations in BD lipid metabolism, validation of differential lipids using targeted lipidomics, and the development of novel diagnostic tools are necessary for a complete understanding of lipid pathways and their application in the diagnosis and improvement of BD treatments.

## Figures and Tables

**Table 1:** Demographic and clinical characteristics of patients and controls were included in the study.

Variable	BD (N = 31)	C (N = 31)
Nationality; race (%)	Serbian; white (100%)	Serbian; white (100%)
Medications use, N (%)		
Antipsychotics of the first generation	2 (6.45%)	/
Antipsychotics of the second generation	24 (77.42%)	/
Anxiolytics	5 (16.13%)	/
Smokers, N(%)	22 (70.97%)	18 (58.06%)
Age range (mean)	20–74 (48.12)	24–54 (47.86)

**Table 2:** Relevant *m*/*z* values selected by the PLS-DA model (combined positive and negative ion modes) relating to the lipids found differently in bipolar disorder (BD) patients and healthy controls (C). * *m*/*z* mass-to-charge ratio; LPC: 1-acyl-sn-glycero-3-phosphocholines; GP: glycero-phospho-lipids; FA: fatty acyls; PA: 1,2-diacyl-sn-glycero-3-phosphates; LPS: 1-acyl-sn-glycero-3-phospho-serines; SP: sphingolipids; Cer: N-acylsphinganines (dihydroceramides); SM: ceramide phospho-cholines (sphingomyelins); PC: diacylglycerophosphocholines; PC O-: 1-alkyl,2-acyl-glycero-phosphocholines; PS: diacylglycerolphosphoserines; DG: diacylglycerols; GL: glycerolipids; TG: triacylglycerols; CE: cholesterol ester; ST, sterol lipids.

No.	Retention Time (min)	VIP Value	VIP Feature Assignment	Measured *m*/*z* *	Ion Mode Adduct	Proposed Formula	Lipid Assignment	Lipid Class
1	1.29	1.5673	LPC 18:2 A	578.3494	[M+OAc]^−^	C_26_H_50_NO_7_P	LPC 18:2	GP
		1.4548	LPC 18:2 A1	520.3385	[M+H]^+^			
		1.3381	LPC 18:2 A2	542.3228	[M+Na]^+^			
2	1.52	1.4205	LPC 16:0 A2	496.3385	[M+H]^+^	C_24_H_50_NO_7_P	LPC 16:0	GP
3	2.15	1.3184	FA 24:0;O	402.3924	[M+NH_4_]^+^	C_24_H_48_O_3_	FA 24:0;O	FA
4	2.15	1.2832	FA 22:0	358.3661	[M+NH_4_]^+^	C_22_H_44_O_2_	FA 22:0	FA
5	3.12	1.0837	PA 25:0	589.3263	[M+K]^+^	C_28_H_55_O_8_P	PA 25:0	GP
6	3.12	1.1387	LPS O-24:1;O	632.3937	[M+Na]^+^	C_30_H_60_NO_9_P	LPS O-24:1;O	GP
7	3.35	1.1909	FA 21:4;O6	415.2349	[M+H]^+^	C_21_H_34_O_8_	FA 21:4;O6	FA
8	4.77	1.1020	Cer 34:1;O2 A	560.5026	[M+Na]^+^	C_34_H_67_NO_3_	Cer 34:1;O2	SP
9	5.09	1.3798	SM 32:1;O2	675.5427	[M+H]^+^	C_37_H_75_N_2_O_6_P	SM 32:1;O2	SP
10	5.17	1.3764	SM 34:2;O2 A1	701.5587	[M+H]^+^	C_39_H_77_N_2_O_6_P	SM 34:2;O2	SP
11	5.43	1.2202	SM 33:1;O2	689.5584	[M+H]^+^	C_38_H_77_N_2_O_6_P	SM 33:1;O2	SP
12	5.63	1.6594	PC 38:6 A	806.5691	[M+H]^+^	C_46_H_80_NO_8_P	PC 38:6	SP
13	5.73	1.9145	PC 36:4	782.5695	[M+H]^+^	C_44_H_80_NO_8_P	PC 36:4	GP
14	5.75	1.2116	SM 34:1;O2 A2	725.5564	[M+Na]^+^	C_39_H_79_N_2_O_6_P	SM 34:1;O2	SP
		1.3527	SM 34:1;O2 A1	703.5748	[M+H]^+^			
15	5.95	1.3059	SM 34:1;O2	703.5746	[M+H]^+^	C_39_H_79_N_2_O6_P_	SM 34:1;O2	SP
16	6.04	1.4245	PC 36:4 B5	782.5716	[M+H]^+^	C_44_H_80_NO_8_P	PC 36:4	GP
17	6.09	1.0887	PC 33:2 B	802.5619	[M+OAc]^−^	C_41_H_78_NO_8_P	PC 33:2	GP
18	6.12	1.7568	PC 34:2 A	816.5779	[M+OAc]^−^	C_42_H_80_NO_8_P	PC 34:2	GP
		1.6487	PC 34:2 A2	1516.1288	[2M+H]^+^			
		1.6482	PC 34:2 A3	796.5254	[M+K]^+^			
		1.6229	PC 34:2 A4	780.5515	[M+Na]^+^			
		1.5566	PC 34:2 A5	758,5787	[M+H]^+^			
		1.1697	PC 34:2 A6	184.0725	[C_5_H_14_NO_4_P+H]^+^			
19	6.37	1.5682	PC O-36:5	766.5744	[M+H]^+^	C_44_H_80_NO_7_P	PC O-36:5	GP
20	6.38	1.0028	PC O-36:4 A1	826.5976	[M+OAc]^−^	C_44_H_82_NO_7_P	PC O-36:4	GP
21	6.46	1.4419	PC O-38:6	792.5894	[M+H]^+^	C_46_H_82_NO_7_P	PC O-38:6	GP
22	6.50	1.8977	PC O-34:3	742.5741	[M+H]^+^	C_42_H_80_NO_7_P	PC O-34:3	GP
23	6.51	1.1837	PC 35:2 A1	772.5849	[M+H]^+^	C_43_H_82_NO_8_P	PC 35:2	GP
24	6.53	1.9588	PC O-36:4	768.5900	[M+H]^+^	C_44_H_82_NO_7_P	PC O-36:4	GP
25	6.60	1.6051	PC O-38:5 A	794.6057	[M+H]^+^	C_46_H_84_NO_7_P	PC O-38:5	GP
26	6.64	2.1701	PC O-34:2	744.5895	[M+H]^+^	C_42_H_82_NO_7_P	PC O-34:2	GP
27	6.73	1.9050	PC O-36:3	770.6046	[M+H]^+^	C_44_H_84_NO_7_P	PC O-36:3	GP
28	6.83	1.2700	PC 35:2	830.5927	[M+H]^+^	C_43_H_82_NO_8_P	PC 35:2	GP
29	6.83	1.8175	PC 36:2 A	844.6088	[M+OAc]^−^	C_44_H_84_NO_8_P	PC 36:2	GP
		1.6528	PC 36:2 A2	808.5828	[M+Na]^+^			
		1.6180	PC 36:2 A3	824.5563	[M+K]^+^			
30	6.87	1.6510	PS 41:4	876.5696	[M+Na]^+^	C_47_H_84_NO_10_P	PS 41:4	GP
31	6.95	1.4782	PC O-32:1	718.5736	[M+H]^+^	C_40_H_80_NO_7_P	PC O-32:1	GP
32	6.97	1.2903	Cer 36:0;O2	568.5651	[M+H]^+^	C_36_H_73_NO_3_	Cer 36:0;O2	SP
33	7.08	1.9935	PC O-40:6	837.6194	[M+NH_4_]^+^	C_48_H_86_NO_7_P	PC O-40:6	GP
34	7.08	1.2704	PC O-34:2	744.5891	[M+H]^+^	C_42_H_82_NO_7_P	PC O-34:2	GP
35	7.11	1.5948	PC O-32:0	720.5892	[M+H]^+^	C_40_H_82_NO_7_P	PC O-32:0	GP
36	7.14	1.4019	PC O-38:5 B	794.6051	[M+H]^+^	C_46_H_84_NO_7_P	PC O-38:5	GP
37	7.22	1.4158	PC O-34:1	746.6050	[M+H]^+^	C_42_H_84_NO_7_P	PC O-34:1	GP
38	7.31	1.6438	PC O-38:4	796.6209	[M+H]^+^	C_46_H_86_NO_7_P	PC O-38:4	GP
39	7.34	1.4761	SM 38:1;O2	759.6370	[M+H]^+^	C_43_H_87_N_2_O_6_P	SM 38:1;O2	SP
40	7.36	1.3589	PC 34:0	762.5997	[M+H]^+^	C_42_H_84_NO_8_P	PC 34:0	GP
41	7.46	1.6474	SM 40:2;O2	785.6529	[M+H]^+^	C_45_H_89_N_2_O_6_P	SM 40:2;O2	SP
42	7.88	1.1853	SM 41:2;O2	799.6680	[M+H]^+^	C_46_H_91_N_2_O_6_P	SM 41:2;O2	SP
43	8.09	1.9568	SM 40:1;O2	787.6687	[M+H]^+^	C_45_H_91_N_2_O_6_P	SM 40:1;O2	SP
44	8.49	1.7303	SM 41:1;O2	801.6839	[M+H]^+^	C_46_H_93_N_2_O_6_P	SM 41:1;O2	SP
45	8.86	2.0248	SM 42:1;O2	815.6997	[M+H]^+^	C_47_H_95_N_2_O_6_P	SM 42:1;O2	SP
46	9.75	1.5408	DG 37:7	647.4579	[M+Na]^+^	C_40_H_64_O_5_	DG 37:7	GL
47	9.96	1.7561	Cer 42:1;O2	672.6257	[M+Na]^+^	C_42_H_83_NO_3_	Cer 42:1;O2	SP
48	11.31	1.0632	TG 52:4 A1	872.7703	[M+NH_4_]^+^	C_55_H_98_O_6_	TG 52:4	GL
		1.0751	TG 52:4 A2	877.7257	[M+Na]^+^			
		1.0570	TG 52:4 A3	893.6996	[M+K]^+^			
49	11.31	1.1380	TG 58:4	956.8632	[M+NH_4_]^+^	C_61_H_110_O_6_	TG 58:4	GL
50	11.49	1.1909	TG 52:3 A1	874.7863	[M+NH_4_]^+^	C_55_H_100_O_6_	TG 52:3	GL
		1.2129	TG 52:3 A2	879.7414	[M+Na]^+^			
		1.0899	TG 52:3 A3	895.7152	[M+K]^+^			
51	11.49	1.2905	TG 58:3	958.8792	[M+NH_4_]^+^	C_61_H_112_O_6_	TG 58:3	GL
52	11.55	1.1582	TG 49:1	836.7694	[M+NH_4_]^+^	C_52_H_98_O_6_	TG 49:1	GL
53	11.58	1.5672	CE 18:2 A1	666.6174	[M+NH_4_]^+^	C_45_H_76_O_2_	CE 18:2	ST
		1.8587	CE 18:2 A2	671.5727	[M+Na]^+^			
		2.0131	CE 18:2 A3	1320.1560	[2M+Na]^+^			
54	11.62	1.4854	TG 48:0 A1	824.7697	[M+NH_4_]^+^	C_51_H_98_O_6_	TG 48:0	GL
55	11.65	1.0435	TG 58:2	960.8947	[M+NH_4_]^+^	C_61_H_114_O_6_	TG 58:2	GL
56	11.79	1.0106	TG 50:0	852.7998	[M+NH_4_]^+^	C_53_H_102_O_6_	TG 50:0	GL

## Data Availability

The data of the lipidomics presented in this study are unavailable due to privacy or ethical restrictions.
